# Mediation Effect of Self-Efficacy and Resilience on the Psychological Well-Being of Lebanese People During the Crises of the COVID-19 Pandemic and the Beirut Explosion

**DOI:** 10.3389/fpsyt.2021.733578

**Published:** 2022-01-10

**Authors:** Jihoon Hong, Hussein Walid Mreydem, Bayan Tarek Abou Ali, Nada Omar Saleh, Sajida Fawaz Hammoudi, Jukab Lee, Junseok Ahn, Jangho Park, Youjin Hong, Sooyeon Suh, Seockhoon Chung

**Affiliations:** ^1^University of Ulsan College of Medicine, Seoul, South Korea; ^2^Faculty of Medical Sciences, Lebanese University, Beirut, Lebanon; ^3^Department of Psychiatry, Ulsan University Hospital, University of Ulsan College of Medicine, Ulsan, South Korea; ^4^Department of Psychiatry, GangNeung Asan Hospital, University of Ulsan College of Medicine, Gangneung, South Korea; ^5^Department of Psychology, Sungshin Women's University, Seoul, South Korea; ^6^Department of Psychiatry, Asan Medical Center, University of Ulsan College of Medicine, Seoul, South Korea

**Keywords:** COVID-19, explosion, psychological, anxiety, stress

## Abstract

**Introduction:** Self-efficacy signifies an individual's belief in their own ability to perform the actions required to achieve a particular performance. In this study, we used an online survey to assess the mediation effect of resilience and self-efficacy on the overall psychological well-being of Lebanese people during the crises of the COVID-19 pandemic and the Beirut explosion.

**Methods:** Overall, 567 Lebanese people participated in an online survey between March 17–28, 2021. The survey included the Stress and Anxiety to Viral Epidemics-6 items (SAVE-6), Patient Health Questionnaire-9 (PHQ-9), Connor–Davidson Resilience Scale-2 items, WHO-5 Well-being Index, General Self-Efficacy scale, and a single item on insomnia. We also assessed their risk perception regarding exposure to COVID-19 or explosions.

**Results:** About 53% of participants were assessed as having depression (PHQ-9 ≥ 10) in the recent crisis. About half of participants (53.2%) reported feeling more stressed by COVID-19 than by the Beirut explosion, and 23.4% felt more stressed by the Beirut explosion than by COVID-19. Only the SAVE-6 score differed significantly between groups with greater stress responses to COVID-19 and the Beirut explosion. Self-efficacy mediated the influence of depression on people's psychological well-being, and self-efficacy and resilience mediated the influence of viral anxiety on psychological well-being.

**Conclusion:** Self-efficacy is important for reducing people's depression and improving their psychological well-being during the Lebanon crises and also mediates the influence of anxiety in response to the viral epidemic on their psychological well-being in some people.

## Introduction

In Lebanon, the health crisis related to the Coronavirus disease 2019 (COVID-19) pandemic occurred during a time when Lebanon was suffering economically, financially, and socially. The first COVID-19 case was reported on February 25, 2020. On March 18, 2020, the Council of Ministers announced the closure of the borders, airport, seaports, and suspension of the affairs of non-essential companies to limit the spread of SARS-CoV-2 (1). These measures led to a limited spread of the emerging virus, and the number of cases did not exceed 20 on most days of March, April, and May (2). Epidemic control continued until the gradual reopening of certain important economic sectors began on April 27, 2020. After this gradual opening, accompanied by the Lebanese expatriates' return, the country again witnessed an increase in the number of people infected with SARS-CoV-2, reaching 166 cases on July 12, 2020 (3).

### COVID-19 and Lockdown in Lebanon

Although the number of COVID-19 cases was increasing, the country's economic status prevented another complete lockdown. As an alternative, areas with a high number of COVID-19 patients were placed in lockdown, while other areas were left open; however, this did not restrict the disease spread. This process continued, despite increases in the number of cases (4). Then, in the Season's Greetings vacation in December, the country opened up without any restrictions. People went out, celebrating without concern for the full hospitals and the ill patients. This led to a marked increase in the number of COVID-19 patients and death toll. On January 8, 2021, the number of cases reached 5,440, which led to an active lockdown on January 14, 2021, where most sectors in the country closed, and the country depended on imports (5).

The pandemic has undoubtedly impacted everyone's life, not only physically but also mentally, as the implemented changes were related not only to the infection itself but also to lifestyle, concerns about physical health, and many other factors. A systematic review and meta-analysis showed that the prevalence of depression, in 14 studies of the general population with a sample size of 44,531, was 33.7%, with a higher rate among women than men, among those with high compared to low education levels, and among middle-aged (20–40 years) compared to older people, due to their concerns about their future (6). Another study on the general population of Mexico reported severe depression symptoms in 27.5%. These symptoms were more severe among women, single people, those without children, those with medical comorbidities, and those with a history of mental health care (7). All these studies showed that COVID-19 caused an increase in the depression prevalence among the general population, given that the pre-pandemic prevalence was around 7.1%. Nevertheless, the findings regarding gender (females more than males) and age groups (more among middle-aged), were similar before and during the pandemic (8).

### The Beirut Explosion in Lebanon

During 2020, other occurrences also had an impact on the marked increase in the number of COVID-19 patients. For instance, on August 4, 2020, the Beirut port explosion occurred, which was one of the most powerful explosions in history. This explosion resulted in more than 200 deaths and more than 5,000 injured people and left ~300,000 homeless (9, 10). Before August 4, 2020, the number of confirmed daily COVID-19 cases mostly remained under 200, except for 221 cases on July 31, 2020. Thereafter, the numbers rapidly increased above this range, possibly because people throughout Lebanon rushed to help and support each other after the explosion, despite the pandemic. Given the ensuing chaos, the country could not enter lockdown at that time (3).

Although the prevalence of depression related to the explosion and its consequences in the Lebanese general population has not been reported, it is likely that numerous people may suffer from depression, similar to a previous study on the effect of an explosion in February 1994 in a full Christian Maronite church in a Beirut suburb (11). The Beirut port explosion significantly impacted the mental health of Lebanese citizens, firstly by depression. This calamity occurred in tandem with a rapidly deteriorating economic and financial crisis, worsened by COVID-19-related lockdown. The situation became unbearable for almost all citizens, demonstrated by an increase in the number of immigrants. Multiple reports have highlighted the collective trauma experienced by survivors of the explosion, including nightmares, flashbacks and fatigue (12), as well as an increased need for mental health consultations (13). Even months before the explosion, an increased prevalence of depression, anxiety, suicides (14) and calls to suicide hotlines was reported (15). This infers a marked rise in all mental health issues post-explosion among the Lebanese population.

### Mediation Effect of Self-Efficacy and Resilience

In such a situation, self-efficacy might help to reduce people's distress. Self-efficacy is the belief that one can perform a behavior or attain specific outcomes (16). It is a strong determinant of human behavior, and it influences the activities in which people choose to engage and how long they persevere in such activities. Self-efficacy signifies an individual's belief in their ability to perform the actions required to achieve a particular performance (16). It is distinct from self-esteem, in that it is a judgment of and belief in the ability, not in the value of an individual's existence, and can be strengthened/deteriorated through experience. Self-efficacy affects choice, performance, and persistence of individuals' behaviors in various areas, such as health (17) or academic performance (18). Prior studies have shown the mediation effect of self-efficacy on general well-being. For instance, according to a study conducted on residents of Stanford Health Care Center, high self-efficacy is associated with better psychological well-being and lower emotional exhaustion (19). In another study of cancer patients, an inverse relationship was observed between self-efficacy and cancer-induced distress. On the other hand, a positive relationship was observed in these patients between self-efficacy and quality of life, or the capacity to cope with stressful situations (20).

Resilience can be defined in various ways. It is a complex phenomenon, which includes personality, interpersonal relationships, and the temporal characteristics of the stressor (21). Resilience means the process of effectively adapting to various adversities and failures, and managing them efficiently (22). It is also the ability to perceive stress as an opportunity, to know one's limitations when it comes to managing stress, fortifying one's resistance to it, and developing a sense of humor (23). Resilient people tend to overcome adversity better, and sometimes accomplish higher outcomes than their original goals. Resilience works as a mediator between depression or anxiety and well-being, according to previous studies. For instance, in a study of 1,419 university students in Hong Kong, more resilient individuals had more positive perceptions, greater satisfaction with life, and lower rates of depression (24). In addition, in a study of frontline nurses in COVID-19 situations, more resilient nurses exhibited less anxiety (25). Another recent study showed that promoting resilient coping styles can reduce the mental burden on patient caregivers (26). Likewise, resilience plays an important role in determining the quality of life as well as coping with emotional distress.

### Aims of the Study

In this study, we assessed the effects of psychological problems on the psychological well-being of Lebanese people during the crises of the COVID-19 pandemic and the Beirut explosion. We also investigated the roles of self-efficacy or resilience as mediators in the relationship between psychological problems caused by both these crises and psychological well-being.

## Methods

### Participants and Procedure

This cross-sectional study was conducted online in Lebanon from March 17, 2021 to March 28, 2021. The survey form was made using Google Forms^®^ (Google LLC, Mountain View, CA), and was distributed by posting an advertisement on the social media network. Adults aged ≥18 years, living in Lebanon were the designated target audience. No financial reward was offered for participation. A total of 567 Lebanese people anonymously and voluntarily responded to this online survey. We excluded the incomplete responses of 67 responders as well as the responses of 94 responders aged <18 years. Finally, 406 responses were included in the analysis. The necessary sample size for this study was calculated to be 384 for a 95% confidence level and a confidence interval of 5. This study protocol was approved and exempted by the Institutional Review Board of the University of Ulsan (2021-R0022-001). The need for obtaining written informed consent was waived in accordance with national legislation and institutional requirements.

The online survey requested information about the respondents' age, sex, marital status, and past psychiatric history or current mood. It also included questions about COVID-19 and the Beirut explosion; for example, “Did you experience being quarantined due to infection with COVID-19?” “Did you experience being infected with COVID-19?” or “Did you experience being physically affected by the Beirut explosion?” In addition, rating scales for symptom assessment were included in the survey form. The survey was developed in Arabic and followed the Checklist for Reporting Results of Internet e-Surveys (CHERRIES) guidelines (27). After its development, the usability and technical functionality of the e-survey form was tested by one of the study investigators, Hussein Walid Mreydem, before its implementation.

### Symptom Assessment

#### Stress and Anxiety to Viral Epidemics-6 Items (SAVE-6)

The SAVE-6 scale is a self-reporting rating scale which was developed to measure the respondent's anxiety response to the COVID-19 pandemic (28). It was derived from the original SAVE-9 scale, in order to measure the work-related stress and anxiety of healthcare workers in response to the COVID-19 pandemic (29). The SAVE-6 scale was validated among the general population in various languages, including Korean (28), Arabic (30), and English (31). In addition, it was reported that the SAVE-6 scale can be reliably applied to special populations, such as medical students (32), public workers (33), and cancer patients (34). Respondents answer each item on a 5-point Likert scale ranging from 0 (never) to 4 (always). Thus, a higher score of the total SAVE-6 scale (range: 0–24) implies severe anxiety to the viral epidemic. In this study, we employed the Arabic SAVE-6 scale (30).

#### Risk Assessment and Perception Regarding the Exposure to COVID-19 or Explosions

We included two single items for assessing Lebanese people's consideration of the risk of exposure to COVID-19 or explosions. We asked participants: “Do you think that you are at higher risk of infection than others?” “Do you think that you are at higher risk of being physically injured by another explosion than others?” and “Currently, is COVID-19 more stressful to you than an explosion?” answered on a 5-point scale (1—strongly disagree, 2—disagree, 3—neutral, 4—agree, 5—strongly agree). A question of “Are you worried that Lebanon will witness another terrorist attack or war in the near future?” was included, to which participants could respond “Yes” or “No.”

#### Patient Health Questionnaire-9 (PHQ-9)

The Patient Health Questionnaire-9 (PHQ-9) is used to measure the severity of a person's depression. Each of the nine items can be rated on a Likert scale (0—not at all to 3—nearly every day), and a high total score (range: 0–27) implies greater symptom severity (0–4, minimal depression; 5–9, mild depression; 10–14, moderate depression; 15–19, moderately severe depression; and ≥20, severe depression) (35). In this study, we applied the Arabic version of the PHQ-9 scale (36).

#### Connor-Davidson Resilience Scale-2 Items (CD-RISC2)

The Connor–Davidson Resilience Scale-2 items (CD-RISC2) scale was developed as a brief measure of resilience. This scale was shortened from the original 25-item CD-RISC scale and contains only two items, item 1 (“Able to adapt to change”), and item 8 (“Tend to bounce back after illness or hardship”). This abbreviated version has been reported to be a reliable rating scale for measuring resilience (37) The two items on the CD-RISC2 scale can be rated on a Likert scale, ranging from 0 (not true at all) to 4 (true nearly all the time). In this study, we used the Arabic version of the CD-RISC2 scale, after receiving permission from the original developer, Dr. Jonathan R. T. Davidson.

#### WHO-5 Well-Being Index

The WHO-5 Well-being Index was developed to measure one's subjective psychological well-being. Each of the five items can be rated on a Likert scale, ranging from 0 (none of the time) to 5 (all the time) (38). The final score is calculated by multiplying the raw total score by 4 (39), and higher score implies greater psychological well-being. We used the Arabic version (40).

#### General Self-Efficacy Scale (GSE)

The General Self-Efficacy (GSE) scale is a 10-item rating scale used to assess one's optimistic self-beliefs regarding coping with stressful events in life (41). The original version included 20 items, but it was subsequently revised to 10 items in 1997. The GSE scale was originally developed in German and has since been developed into other languages (42). Each of four items can be rated on a Likert scale ranging from 1 (not at all true) to 4 (absolutely true). The total score ranges from 10 to 40, with higher scores reflecting a higher level of self-efficacy. In this study, we employed the Arabic version of the GSE scale, available on the GSE website (43).

#### Single Item of Insomnia

We also included a single item intended to measure the quality of sleep, “How is your sleep quality?” (0, very good, to 10, very poor) as a brief measure of sleep quality.

### Statistical Analysis

We conducted the statistical analysis using the SPSS version 21.0 for Windows (IBM Corp., Armonk, NY) and JASP 0.14.1. Clinical characteristics were summarized as mean ± standard deviation, and the level of significance was defined as two-tailed *p* < 0.05. Spearman's rank-order correlation analysis was used to examine the association among age and rating scales scores, as the distribution of the PHQ-9 scores was not within the normal distribution. One-way analysis of variance with Scheffe's *post-hoc* analysis was used to explore differences in clinical variables and rating scales scores, except for the PHQ-9, which was done using the Kruskal-Wallis test, among groups defined as those who found the COVID-19 pandemic more stressful (the COVID-19 group), those who were neutral (neutral group), and those who found the Beirut explosion more stressful (Beirut explosion group). Chi-square analysis was used to examine the differences in categorical variables among groups. Linear regression analysis was used to examine the influence of each rating scale (PHQ-9, SAVE-6, CD-RISC2, or GSE) on psychological well-being. Finally, to explore the mediating effect of self-efficacy on the relationship of depression with psychological well-being, a bootstrap method with 2,000 resamples was implemented among all participants. After that, a bootstrap method with 2,000 resamples was implemented again among participants who were more stressful to COVID-19 and those who were more stressful to the Beirut explosion separately.

## Results

### Clinical Characteristics of Participants

All of 406 subjects aged ≥18 years old were finally included in the analysis, and 82.0% were female, 59.1% were single, and their mean age was 29.3 ± 12.5 years old (Table 1). They live in Beirut (*n* = 54, 13.3%), Mount Lebanon (*n* = 45, 11.1%), North (*n* = 21, 5.2%), Akkar (*n* = 8, 2.0%), South (*n* = 24, 5.9%), Nabatieh (*n* = 15, 3.7%), Beqaa (*n* = 228, 56.2%), and Baalbek-Hermel (*n* = 11, 2.7%). About the questions related to the COVID-19 and the Beirut explosion, 53.7% have experienced being quarantined, 36.7% being infected, and 1.2% being physically damaged. All of 125 (30.8%) responded that they have past psychiatric history, and 55.4% answered that they have currently psychiatric symptoms that need to be helped. About the risk perception, 20.2% of participants answered that they thought they were at higher risk of COVID-19 infection than others, and 13.8% answered that they though that they were at higher risk of being physically injured by another explosion than others. And also, 83.5% worried that Lebanon will witness another terror or war in the near future.

**Table 1:** Demographic characteristics of participants (*N* = 406).

**Variables**	**Mean ± SD, *N* (%)**
**Sex (female)**	333 (82.0%)
**Age, years old**	29.3 ± 12.5
**Marital status**	
Single	240 (59.1%)
Married, without children	14 (3.4%)
Married, with children	136 (33.5%)
**Questions on COVID-19 and the Beirut explosion**
Did you experience being quarantined due to infection with COVID-19? (Yes)	218 (53.7%)
Did you experience being infected with COVID-19? (Yes)	149 (36.7%)
Did you experience being physically damaged by the Beirut explosion? (Yes)	5 (1.2%)
**Psychiatric history**	
Have you had experience of or were you treated for depression, anxiety, or insomnia? (Yes)	125 (30.8%)
At the moment, do you think you are depressed or anxious, or do you need help for your mood state? (Yes)	225 (55.4%)
**Risk perception**	
Do you think that you are at higher risk of infection than others? (Agree^*^)	82 (20.2%)
Do you think that you are at higher risk of being physically injured by another explosion than others? (Agree^*^)	56 (13.8%)
Currently, is COVID-19 more stressful to you than an explosion? (Agree^*^)	216 (53.2%)
Currently, is the explosion more stressful to you than COVID-19? (Agree^*^)	95 (23.4%)
Are you worried that Lebanon will witness another terrorist attack or war in the near future? (Yes)	339 (83.5%)
**Rating scales**	
Patient Health Questionnaire-9 items	10.6 ± 5.8
PHQ-9 ≥ 10	216 (53.2%)
Stress and Anxiety to Viral Epidemics-6 items	11.7 ± 4.4
WHO-5 well-being index	44.3 ± 24.6
Connor-Davidson Resilience Scale 2 items	4.9 ± 1.8
Single item—quality of sleep	5.1 ± 2.8
General self-efficacy	27.2 ± 6.3

* *“Agree” is the responses of “agree” and “strongly agree”*.

Spearman's correlation analysis (Table 2) showed that older age was significantly correlated with a low level of depression (PHQ-9, rho = −0.19, *p* < 0.01) and anxiety related to the viral epidemic (SAVE-6, rho = −0.12, *p* = 0.014), and higher self-efficacy (GSE, rho = 0.12, *p* = 0.014) and psychological well-being (WHO-5, rho = 0.21. *p* < 0.001) levels. The PHQ-9 scale score was correlated with a high score on the SAVE-6 (rho = 0.38, *p* < 0.001), and lower scores of the WHO-5 (rho = −0.55, *p* < 0.001), CD-RISC2 (rho = −0.29, *p* < 0.001), and GSE (rho = −24, *p* < 0.001), and poor sleep quality (rho = 0.31, *p* < 0.001). The SAVE-6 score was significantly correlated with low scores on the WHO-5 (rho = −0.24, *p* < 0.001), CD-RISC2 (rho = −0.29, *p* < 0.001), and GSE (rho = −0.24, *p* < 0.001), and poor sleep quality (rho = 0.17, *p* < 0.001). The psychological well-being significantly correlated with higher scores on the CD-RISC2 (rho = 0.29, *p* < 0.001) and GSE (rho =0 .30, *p* < 0.001), and better sleep quality (rho = −0.22, *p* < 0.001). The CD-RISC2 scale score was significantly correlated with good sleep quality (rho = −0.13, *p* = 0.012) and a high GSE score (rho = 0.41, *p* < 0.001).

**Table 2:** Spearman's correlation coefficients of each variable in all subjects (*n* = 406).

**Variables**	**Age**	**PHQ-9**	**SAVE-6**	**WHO-5**	**CD-RISC2**	**Quality of sleep**	**GSE**
Age	1.000						
PHQ-9	−0.19^**^	1.000					
SAVE-6	−0.12^*^	0.38^**^	1.000				
WHO-5	0.21^**^	−0.55^**^	−0.24^**^	1.000			
CD-RISC2	0.03	−0.29^**^	−0.29^**^	0.29^**^	1.000		
Quality of sleep	−0.01	0.31^**^	0.17^**^	−0.22^**^	−0.13^*^	1.000	
GSE	0.12^*^	−0.24^**^	−0.21^**^	0.30^**^	0.41^**^	−0.09	1.000

*PHQ-9, Patient Health Questionnaire-9; SAVE-6, Stress and Anxiety to Viral Epidemics - 6 items; WHO-5, World Health Organization-5 well-being index; CD-RISC 2, Connor-Davidson Resilience Scale 2 items; GSE, General Self-Efficacy*.

** *p < 0.01*.

* *p < 0.05*.

### Stressful to COVID-19 and Beirut Explosion

Among respondents, 216 (53.2%) participants reported that they feel more stressful to the COVID-19, in contrast that 95 (23.4%) participants feel stressful to the Beirut explosion (Table 3). There was no significant difference in age, sex, marital status, and responses to the questions on risk perceptions. However, among responses to questions on COVID-19 and the Beirut explosion, the proportion of participants who experienced being quarantined due to COVID-19 was significantly higher among those more stressed about COVID-19 and those more stressed about the explosion than among those in the neutral group (*p* = 0.008). The proportion of participants who were feeling depressed or anxious or needed help for their mood state were higher in the COVID-19 and Beirut explosion groups than in the neutral group (*p* = 0.043). The PHQ-9 scale score was significantly higher in the COVID-19 than in the neutral group (*p* = 0.015). The SAVE-6 scale score was significantly higher in those more stressed by COVID-19 than among those who were neutral or more stressed by the Beirut explosion (*p* < 0.001). The CD-RICS-2 scale score was significantly lower in those more stressed by COVID-19 than in the neutral group (*p* = 0.025).

**Table 3:** Comparison of demographic variables and rating scales scores among subjects grouped based on the responses to the question of “now, is COVID-19 more stressful than an explosion to you?” (*N* = 406).

**Variables**	**More stressful to COVID-19 (*N* = 216)**	**Neutral (*N* = 95)**	**More stressful to explosion (*N* = 95)**	***P*-value**
**Sex (female)**	177 (81.9%)	73 (76.8%)	83 (87.4%)	0.168
**Age**	29.3 ± 11.7	29.9 ± 14.6	28.5 ± 11.9	0.739
**Marital status (single)**	123 (51.3%)	59 (64.1%)	58 (63.7%)	0.657
**Questions on COVID-19 and the Beirut explosion**				
Did you experience being quarantined due to infection with COVID-19? (Yes)	123 (56.9%)	38 (40.0%)	57 (60.0%)	0.008^**^
Did you experience being infected with COVID-19? (Yes)	83 (38.4%)	26 (27.4%)	40 (42.1%)	0.081
Did you experience being physically damaged by the Beirut explosion? (Yes)	1 (0.5%)	1 (1.1%)	3 (3.2%)	–
**Psychiatric history**				
Have you had experience of or were you treated for depression, anxiety, or insomnia? (Yes)	65 (30.1%)	24 (25.3%)	36 (37.9%)	0.160
At the moment, do you think you are depressed or anxious, or do you need help for your mood state? (Yes)	127 (58.8%)	42 (44.2%)	56 (58.9%)	0.043^**^
**Risk perception**				
Do you think that you are at higher risk of infection than others? (Agree^*^)	49 (22.7%)	19 (20.0%)	14 (14.7%)	0.224
Do you think that you are at higher risk of being physically injured by another explosion than others? (Agree^*^)	35 (16.2%)	8 (8.4%)	13 (13.7%)	0.186
Are you worried that Lebanon will witness another terrorist attack or war in the near future? (Yes)	187 (86.6%)	72 (75.8%)	80 (84.2%)	0.060
**Rating scales**				
Patient Health Questionnaire-9 items	11.3 ± 5.9	9.3 ± 5.4	10.4 ± 5.9	**0.015** ^†^ ^***^
PHQ-9 ≥ 10	130 (60.2%)	39 (41.1%)	47 (49.5%)	0.006^***^
Stress and Anxiety to Viral Epidemics-6 items	12.6 ± 4.4	10.7 ± 4.5	10.6 ± 4.1	<0.001^****^
WHO-5 well-being index	41.9 ± 24.5	49.1 ± 24.6	44.9 ± 24.5	0.061
Connor-Davidson Resilience Scale 2 items	4.7 ± 1.7	5.2 ± 1.5	5.1 ± 2.1	0.025^*****^
Single item—quality of sleep	5.2 ± 2.8	5.2 ± 2.7	4.7 ± 2.7	0.319
General Self-Efficacy	27.1 ± 6.3	27.2 ± 6.6	27.3 ± 6.2	0.975

† *Kruskal-Wallis test was done*.

* *“Agree” is the responses of “agree” and “strongly agree”*.

** *COVID-19 = Beirut explosion > neutral*.

*** *COVID-19 > neutral*.

**** *COVID-19 > neutral = Beirut explosion*.

***** *COVID-19 < neutral*.

*Bold value indicates significant level*.

**Figure 1:**
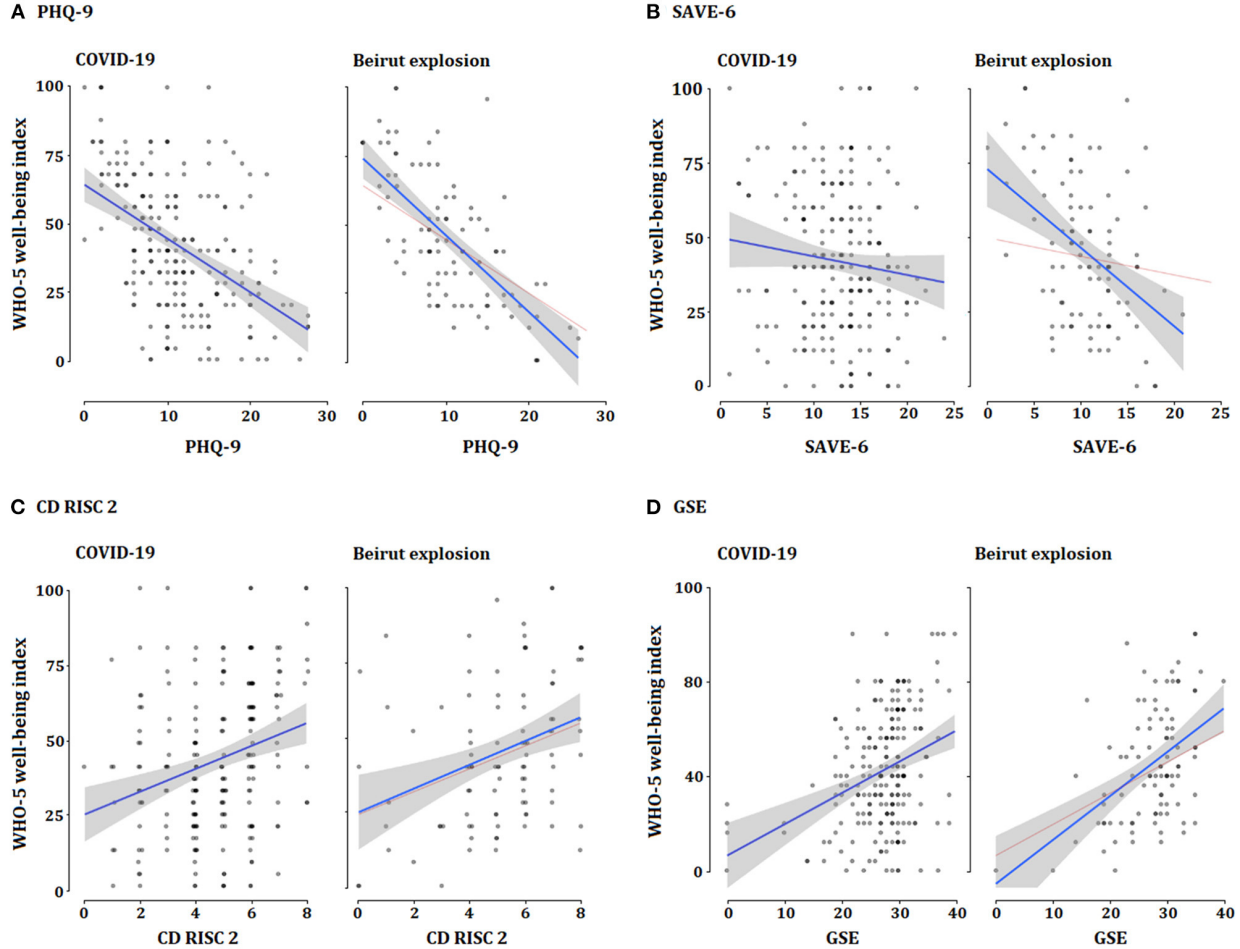
Linear regression analysis of psychological well-being with each rating scale among subjects were answered feeling more stressful to COVID-19 or Beirut explosion. **(A)** Depression and psychological well-being. **(B)** Viral anxiety and psychological well-being. **(C)** Resilience and psychological well-being. **(D)** Self-efficacy and psychological well-being.

Between the COVID-19 vs. Beirut explosion, only the SAVE-6 score, among all demographic variables and rating scale scores, was significantly different. The SAVE-6 score was significantly higher among participants more stressed about COVID-19 (12.6 ± 4.4) than among those more stressed about the Beirut explosion group (10.6 ± 4.1) [*t*_(309)_ = 3.865, *p* < 0.01). Linear regression analysis revealed that the psychological well-being of Lebanese people was associated with depression in those more stressed by COVID-19 (ß = −0.47, *p* < 0.001) and by the Beirut explosion (ß = −0.68, *p* < 0.001, Figure 1). Moreover, psychological well-being was associated with resilience or self-efficacy in both the COVID-19 (CD-RISC2, ß = 0.92, *p* < 0.001; GSE, ß = 0.34, *p* < 0.001) and the Beirut explosion (CD-RISC2, ß = 0.34, *p* < 0.001; GSE, ß = 0.47, *p* < 0.001) groups. However, the SAVE-6 score was associated with psychological well-being only in the Beirut explosion group (ß = −0.44, *p* < 0.001), and not in COVID-19 group (ß = −0.11, *p* = 0.11).

### Mediation Effect of Self-Efficacy or Resilience on the Association Between Depression or Viral Anxiety and Psychological Well-Being

Mediation analysis showed that the complete pathway from depression (independent variable) to self-efficacy (mediator) to psychological well-being of Lebanese people (dependent variable) was significant (Table 4) among all participants, indicating that self-efficacy partially mediates the effects of depression on psychological well-being. The mediating effect of self-efficacy was still observed among subgroup of participants who were more stressful to COVID-19, but the effect was not observed among participants who were more stressful to the Beirut explosion. Resilience does not mediate the association between depression and psychological well-being in this sample.

**Table 4:** The mediation effect of self-efficacy on the relationship between depression and psychological well-being.

**Effect**	**Standardized estimator**	**S.E**.	***Z*-value**	***p***	**95% CI**
**A) Among all participants (*****N*** **=** **406)**					
Direct effect:					
PHQ-9 → WHO-5	−0.48	0.04	−11.20	<0.001	−0.56 ~−0.39
Indirect effect:					
PHQ-9 → GSE → WHO-5	−0.05	0.01	−3.29	0.001	−0.07 ~−0.02
PHQ-9 → CD-RISC2 → WHO-5	−0.02	0.01	−1.44	0.15	−0.05 ~ 0.01
Total effect:					
PHQ-9 → WHO-5	−0.54	0.04	−12.94	<0.001	−0.62 ~−0.46
**B) Among participants more stressful to COVID-19 (*****N*** **=** **216)**					
Direct effect:					
PHQ-9 → WHO-5	−0.41	0.06	−6.89	<0.001	−0.53 ~−0.29
Indirect effect:					
PHQ-9 → GSE → WHO-5	−0.04	0.02	−2.02	0.04	−0.08 ~−0.001
PHQ-9 → CD-RISC2 → WHO-5	−0.03	0.02	−3.39	0.13	−0.06 ~ 0.01
Total effect:					
PHQ-9 → WHO-5	−0.47	0.06	−7.87	<0.001	−0.59 ~−0.36
**C) Among participants more stressful to the Beirut explosion (*****N*** **=** **95)**					
Direct effect:					
PHQ-9 → WHO-5	−0.59	0.08	−7.17	<0.001	−0.76 ~−0.43
Indirect effect:					
PHQ-9 → GSE → WHO-5	−0.07	0.05	−1.43	0.15	−0.17 ~ 0.03
PHQ-9 → CD-RISC2 → WHO-5	−0.02	0.03	−0.61	0.54	−0.08 ~ 0.04
Total effect:					
PHQ-9 → WHO-5	−0.68	0.08	−9.07	<0.001	−0.83 ~−0.54

*S.E., standard error; CI, confidence interval; PHQ-9, Patient Health Questionnaire-9 items; WHO-5, World Health Organization-5 Well-being index; GSE, General Self-Efficacy; CD-RISC2, Connor Davidson Resilience Scale 2 items*.

The influence of viral anxiety on psychological well-being was mediated by self-efficacy and resilience among all participants (Table 5). Among participants who were more stressful to COVID-19, the direct effect of viral anxiety on psychological well-being was not observed. However, resilience and self-efficacy fully mediated the association. Among participants who reported to be more stressful to the Beirut explosion, self-efficacy mediated the influence of viral anxiety on psychological well-being.

**Table 5:** The mediation effect of resilience or self-efficacy on the relationship between viral anxiety and psychological well-being.

**Effect**	**Standardized estimator**	**S.E**.	***Z*-value**	***p***	**95% CI**
**A) Among all participants (*****N*** **=** **406)**					
Direct effect:					
SAVE-6 → WHO-5	−0.18	0.05	−3.85	<0.001	−0.28 ~−0.09
Indirect effect:					
SAVE-6 → GSE → WHO-5	−0.05	0.02	−3.17	0.002	−0.08 ~−0.02
SAVE-6 → CD-RISC2 → WHO-5	−0.04	0.02	−2.43	0.015	−0.07 ~−0.01
Total effect:					
SAVE-6 → WHO-5	−0.27	0.05	−5.65	<0.001	−0.37 ~−0.18
**B) Among participants more stressful to COVID-19 (*****N*** **=** **216)**					
Direct effect:					
SAVE-6 → WHO-5	−0.02	0.07	−0.24	0.81	−0.14 ~ 0.13
Indirect effect:					
SAVE-6 → GSE → WHO-5	−0.04	0.02	−2.04	0.04	−0.09 ~−0.002
SAVE-6 → CD-RISC2 → WHO-5	−0.05	0.02	−2.34	0.02	−0.09 ~−0.01
Total effect:					
SAVE-6 → WHO-5	−0.11	0.07	−1.64	0.10	−0.24 ~ 0.02
**C) Among participants more stressful to the Beirut explosion (*****N*** **=** **95)**					
Direct effect:					
SAVE-6 → WHO-5	−0.32	0.09	−3.45	<0.001	−0.50 ~−0.14
Indirect effect:					
SAVE-6 → GSE → WHO-5	−0.12	0.05	−2.29	0.02	−0.21 ~ 0.02
SAVE-6 → CD-RISC2 → WHO-5	−0.01	0.03	−0.21	0.83	−0.08 ~ 0.06
Total effect:					
SAVE-6 → WHO-5	−0.44	0.09	−4.73	<0.001	−0.62 ~−0.26

*S.E., standard error; CI, confidence interval; SAVE-6, Stress and Anxiety to Viral Epidemics-6 items; WHO-5, World Health Organization-5 Well-being index; GSE, General Self-Efficacy; CD-RISC2, Connor Davidson Resilience Scale 2 items*.

## Discussion

In this study, 53.2% of participants were assessed as having depression (PHQ-9 ≥ 10) in the recent crisis. About half of participants (53.2%) reported feeling more stressed by COVID-19 than by the Beirut explosion, and 23.4% felt more stressed by the Beirut explosion than by COVID-19. The proportion of participants rated as having depression was marginally significantly higher in the COVID-19 than in the Beirut explosion group (*p* = 0.079). Among participants, in the COVID-19 group, psychological well-being was not significantly associated with the level of anxiety about the viral epidemic. Conversely, among participants in the Beirut explosion group, better psychological well-being was significantly associated with lower levels of anxiety about the viral epidemic. Self-efficacy partially mediated the effects of depression on psychological well-being, and the influence of viral anxiety on psychological well-being was mediated by self-efficacy and resilience among all participants.

### Depression, Psychological Well-Being, and Mediation Effect of Self-Efficacy

In this study, the proportion of subjects who were feeling depressed or anxious or needed help for their mood state was significantly higher among those in the COVID-19 and Beirut explosion groups than among those in the neutral group. The PHQ-9 scale score was significantly higher in the COVID-19 group than in the neutral group. In 2020, the COVID-19 pandemic exacerbated Lebanon's economic problems and compounded poverty. The economic collapse and the concurrent pandemic affected the medical sector markedly. The explosion paralyzed the Beirut seaport, which handles around 70% of the country's imports, which affected the food supply considerably, as Lebanon imports 85% of its food (44).

We observed that the self-efficacy of Lebanon people mediated the influence of depression on their psychological well-being. Self-efficacy plays a role in reducing depression in the pandemic era (45–47). Self-efficacy may also influence one's vigilance toward potential threats. People with high self-efficacy believe that they can control these threats, but people with low self-efficacy may overestimate the threats (48). A study of the importance of coping self-efficacy was conducted on 27 victims of the Oklahoma explosion, to explore psychological pain after the explosion (49). The study identified the effects of coping self-efficacy 2 months as well as 1 year after the explosion. Two months after the explosion, individual coping self-efficacy was found to have had a significant impact on the victims' general trauma-related perception, even after controlling for several factors, including income, social support, the threat of death, and loss of resources. In a survey conducted 1 year later, coping self-efficacy also had a significant impact on the general trauma-related distribution. In another study of 97 veterans, the correlations among self-efficacy, combat exposure, and post-traumatic stress disorders (PTSD) and depression were identified. Self-efficacy also played a role in controlling the relationship between combat exposure and PTSD (50). In a study conducted in 2005, related to the September 11, 2001, terrorist attacks, self-enhancement was related to resilience and to lower levels of depression and PTSD (51). These results provide the information that it is needed to consider including self-efficacy skill-building as part of the psychological support system for victims.

In this study, however, the mediation effect of self-efficacy on the influence of depression on psychological well-being was not observed among participants who were more stressful to the Beirut explosion. One possible explanation is that the Beirut explosion occurred in August, 2020, but the survey was conducted in March, 2021. The recall bias might affect the results among participants who reported that they were more stressful to the Beirut explosion.

### Viral Anxiety, Psychological Well-Being, and Mediation Effect of Self-Efficacy and Resilience

The proportion of subjects who had experienced being quarantined was significantly higher among those more stressed about COVID-19 and by the explosion group than among the neutral group. The SAVE-9 scale score was significantly higher in the COVID-19 group than in the neutral and Beirut explosion groups. It is reasonable that the level of anxiety related to the viral epidemic, linked to participants' experience of being quarantined, may be higher among those more stressed about COVID-19. According to a study of people who were quarantined during the COVID-19 pandemic, quarantined people showed high stress, anxiety, and depression (52). Other studies comparing people who were quarantined in affected areas, people quarantined in unaffected areas, and people who were not quarantined reported that people who were quarantined showed more symptoms of depression and anxiety than those who were not quarantined (53).

Interestingly, the relationship between the SAVE-6 score on Lebanese people's psychological well-being was observed only among those more stressed about the Beirut explosion, but not in those more stressed about COVID-19 from univariate regression analysis (Figure 1B). In addition, the level of the SAVE-6 score was significantly higher among those more stressed by COVID-19 than about the Beirut explosion (Table 3). Thus, there is a higher level of anxiety about the viral epidemic in Lebanese people who feel stressed about the Beirut explosion, which might influence their psychological well-being while they are stressed about the Beirut explosion. The high level of viral anxiety among participants who feel more stressed about COVID-19 was not associated with psychological well-being, which may be due to the relatively higher SAVE-6 score.

In this study, resilience mediated the influence of viral anxiety on psychological well-being among all participants. In COVID-19 pandemic, people's high level of resilience was reported to related with low level of stress and high level of psychological well-being among the general population (54), even in special population such as public workers (55), schoolteachers (56), or healthcare workers (57). In this sample, however, we could not observe mediating effect of resilience among participants who were more stressful to Beirut explosion. Similar to the lack of mediating effect of self-efficacy in this sample, the recall bias may influence the results among participants who reported that they were more stressful to the Beirut explosion.

There were some methodological weaknesses in this study. The proportion of participants who experienced a perceived risk and felt more stressed about the Beirut explosion was relatively lower (13.8 and 23.4%, respectively) than expected, while the proportion of participants who were more stressed about the COVID-19 pandemic was relatively high (53.2%). The Beirut explosion was determined to be due to 2,750 tons of ammonium nitrate that had been stored unsafely at a warehouse in the port, rather than a terrorist attack (58). Therefore, this type of explosion is unlikely to appear again easily in the near future. We need to consider the difference in the responses regarding the Beirut explosion and another terrorist attack or war, as 83.5% of respondents answered that they worried that Lebanon would witness terrorism or war in the near future. Only 54 (13.3%) respondents were staying in Beirut. Although data was not shown and the sample sizes were uneven, the ratios of responses in the COVID-19 and the Beirut explosion groups were significantly higher (*p* = 0.007) among participants living in Beirut (*N* = 54, COVID-19: 33.3%; neutral: 33.3%; Beirut explosion: 33.3%) than in other regions (*N* = 352, COVID-19: 56.3%; neutral: 21.9%; Beirut explosion: 21.8%). After the port blast, many citizens of Beirut left their devastated houses and workplaces and moved to nearby villages, but the number of people still living in Beirut remains higher than the number who left. Most of our participants were not living in Beirut, which may explain the results obtained. Living in Beirut, particularly near the blast, being injured during the blast, or knowing somebody who died during the blast is likely to make a person more anxious and depressed than a person not living in Beirut, who had not been injured, does not know anyone killed during the blast. This should be studied further by focusing only on Beirut city and particularly on people from areas damaged by the blast.

Moreover, in this study, we did not interview the victims of the Beirut explosion who had been physically injured. Only five (1.2%) participants answered that they were physically injured by the Beirut explosion, while 53.7% had been quarantined and 36.7% had been infected with COVID-19. This may cause mistaken minimization of the psychological trauma of the Beirut explosion. However, the aim of this study was not to compare the psychological symptoms between COVID-19 infected patients and the Beirut explosion victims directly, but rather to explore the psychological state of the general population living in Lebanon during the concurrent crisis of the COVID-19 pandemic and the Beirut explosion.

This study had some limitations. The first major limitation was that the time frames between each of the two crises and the survey were very different. The Beirut explosion occurred in August, 2020, but the survey was conducted in March, 2021. Therefore, recall bias might have influenced the respondents' answers regarding this event. To address this limitation, we assessed the respondents' perception of the risk of and the level of stress in response to each event, even though we could not measure the exact direct hazards from the Beirut explosion. Second, it was conducted via an anonymous online survey, as face-to-face interviews were not possible due to the possible risk of viral spread. Third, the female preponderance (82.0%) may influence the results of this study. In this pandemic, depression or mental distress was reported to be higher among women in the general population (59), as well as in special populations, such as healthcare workers. Fourth, the small proportion of participants who are staying in Beirut may cause a misunderstanding of the real psychological status related to COVID-19 and the Beirut explosion simultaneously. Fifth, the single item question for sleep quality was not formally validated; a more reliable scale for sleep quality needs to be employed in future studies. Similarly, a psychological trauma-related rating scale might have been useful for assessing psychological well-being during these crises, and must be considered for future studies. Furthermore, there was a marked imbalance between the number of participants who experienced physical injury from the Beirut explosion compared to those who had experienced being quarantined or infected. The sample of respondents for our survey was not homogeneous, since only a few of them had experienced the Beirut explosion. This is likely to have caused a sampling bias.

### Conclusions

This study explored the influence of COVID-19 and a physical disaster (the Beirut explosion) on people's psychological well-being, and the mediating effect of their self-efficacy, which has not been reported previously. We observed that self-efficacy partially mediated the effects of depression on psychological well-being, but the effect was not observed among participants who were more stressful to the Beirut explosion. The influence of viral anxiety on psychological well-being was mediated by self-efficacy and resilience among all participants, though the effect was not observed among some participants, dependently on their stressful feeling to COVID-19 or Beirut explosion. We hope that our findings can help to develop a psychological support system for the Lebanese people during this crisis.

## Data Availability Statement

The raw data supporting the conclusions of this article will be made available by the authors, without undue reservation.

## Ethics Statement

The studies involving human participants were reviewed and approved by Institutional Review Board of the University of Ulsan (2021-R0022-001). Written informed consent for participation was not required for this study in accordance with the national legislation and the institutional requirements.

## Author Contributions

SC and SS: conceptualization and investigation. HM, BA, NS, and SH: data curation. SC: formal analysis and supervision. SC, JP, JL, JA, and YH: methodology. JH: visualization. JH, HM, and SC: writing—original draft. All authors writing—review and editing.

## Funding

This work was supported under the Framework of International Cooperation Program managed by the National Research Foundation of Korea (FY2020K2A9A1A01094956).

## Conflict of Interest

The authors declare that the research was conducted in the absence of any commercial or financial relationships that could be construed as a potential conflict of interest.

## Publisher's Note

All claims expressed in this article are solely those of the authors and do not necessarily represent those of their affiliated organizations, or those of the publisher, the editors and the reviewers. Any product that may be evaluated in this article, or claim that may be made by its manufacturer, is not guaranteed or endorsed by the publisher.
